# Socially connected while apart: the use of technology to increase social connection between nursing home residents and their relatives

**DOI:** 10.3389/fpubh.2024.1296524

**Published:** 2024-01-25

**Authors:** Sehrish Andleeb Akhtar

**Affiliations:** Faculty of Social Sciences, Department of Social Work, Child Welfare and Social Policy, Oslo Metropolitan University, Oslo, Norway

**Keywords:** communication technology, social connectedness, social isolation, nursing home, eldercare, long-term care

## Abstract

**Introduction:**

This study examines whether the use of a communication technology can enhance social connectedness among nursing home residents in Norway. The concept of social connectedness suggest that positive and significant interpersonal relationships can help reduce the risk of loneliness and social isolation among older adults and can be beneficial for both their health and overall well-being. In recent years, technology has been increasingly utilized as a method to overcome physical distances and to keep families connected. Although the use of digital solutions among older people has increased in recent years, few studies have addressed the use and impact of such technologies within a nursing home context.

**Methods:**

A total of 225 residents participated in the study, making it one of the few quantitative studies that examine the use of communication technologies in nursing homes at such a large scale. The study combines two sources of data: (1) survey data collected over a 14-month period, in three different waves, at all public nursing homes in Oslo municipality and (2) a highly detailed weekly datastream from each Komp-device, which provides an objective measure of the frequency of contact between the residents and their families. The two sources of data were combined and analyzed using multiple regression analysis.

**Results:**

The regression analysis revealed a positive and significant relationship between Komp use and increased social satisfaction among the residents. The results indicate that Komp is a feasible communication tool to help nursing home residents maintain relations with their families.

**Discussion:**

The positive response to Komp among the residents suggests that when designed with the user’s needs in mind, technology can indeed facilitate meaningful social interactions, even for those with limited technological experience. Such interventions can thus be crucial in bridging the gap between older residents and the outside world, effectively addressing their unique challenges of social isolation and disconnection from the broader community.

## 1 Introduction

Good social connections are fundamental for our overall health and well-being. A lack of social interaction can contribute to loneliness and social isolation (1), and can increase the risk of depression and anxiety among older adults (2). Social isolation in later life have also been linked to somatic health issues such as coronary heart disease and stroke, increased risk of mortality, and cognitive decline (3–5). While age does not independently cause social isolation or loneliness, age-related experiences such as retirement, living alone, loss of friends and family, and loss of health and mobility can exacerbate the risk of social isolation and loneliness (6–8).

Residents of long-term care facilities tend to face an increased risk of loneliness and social isolation (6, 9). A study of Norwegian nursing homes found that residents tend to experience sadness and loneliness as a result of a lack of meaningful communication with other residents and the nursing staff (10). Although long-term care facilities provide their residents with a wide range of services to assist with their medical and non-medical needs, they frequently fall short on providing sufficient social and emotional care (9, 11). Most institutionalized older adults are therefore dependent on family members and friends to uphold their social well-being. However, maintaining regular contact can be a challenge for family members and friends as well, as they may be hindered by personal commitments and geographical distances (6).

Technological solutions such as video calls and social media have previously been associated with decreased feelings of depression and loneliness, as well as an overall increase in quality of life for older adults (12–15). Given their potential to foster social connection from afar, social technologies developed to connect older relatives with their families could be the interventions needed to bridge the gap between long-term care residents and the outside world. However, research on implementation and use of tailored technologies at long-term care facilities is scant due to access, recruitment, and ethical challenges with this population (16, 17). Little is therefore known about the impact of such technologies among institutionalized older adults.

This study seeks to address this gap by examining the use of a social technology called Komp in all public nursing homes in Norway. Komp is a communication solution developed specifically to connect older adults with their families to combat social isolation. Komp has no touchscreen and is designed specifically for older adults with little to no digital experience, making it a feasible device to be implemented in long-term care. I use comprehensive data to examine (1) whether the communication technology Komp can facilitate social contact between Norwegian nursing home residents and their families, and (2) whether the use of Komp can increase the residents’ overall satisfaction with their social contact.

## 2 Background

### 2.1 Why social connectedness matters

The degree and quality of our interactions, relationships and engagement with other people is often referred to as *social connectedness*. Connectedness is considered to be a fundamental human need, and it encompasses a sense of belonging and attachment that people experience when they are part of a supportive social environment (18, 19). When our need for connectedness remains unfulfilled it can have a negative impact on our health and overall well-being (20). The health risks can be particularly severe for older adults as they are more likely to face stressful life course transitions, health problems and mobility issues (7).

Social isolation among older adults has been a central concern in research and public health for many years. Although conceptualizations vary, social isolation is typically defined as an objective lack of social contacts and infrequent interactions (6, 7). Cornwell and Waite (7) build upon a body of literature within the disciplinary approaches of sociology and psychology and distinguish between two forms of social isolation: *social disconnectedness* and *perceived isolation.* Social disconnectedness is characterized by a lack of contact with others, and includes indicators such as a small social network, infrequent social interactions, and low levels of participation in social activities and groups. Perceived isolation, on the other hand, is a subjective experience of shortcomings in one’s relationships compared to what one would like to have, and it includes feelings of loneliness and lack of belonging. This paper builds upon the distinction presented by Cornwell and Waite to contextualize the social outcomes addressed in this study.

### 2.2 Risk of disconnectedness and perceived isolation in long-term care facilities

Long-term care facilities foster a social environment through a variety of means, including shared living spaces, communal dining areas, group activities, volunteer involvement and daily interactions with staff (10, 21). While such means could potentially help address many of the risk factors that are typically associated with social isolation, researchers have identified several barriers that can hinder the full realization of this potential.

As a heterogeneous group, residents of long-term care vary in terms of their characteristics, needs, and backgrounds. Various aspects at the individual level, such as language barriers, cultural differences, differences in life experiences and age differences can influence the level of connection residents may have with one another. Older residents also tend to have complex health problems including sensory, cognitive or mobility impairments which can impact their opportunities to establish or maintain social connections (6, 10, 22). In a scoping review by Boamah et al. (6), communication barriers and cognitive impairment were identified as two key factors that could increase the risk of social isolation in long-term care. Residents with cognitive impairments may struggle to remember new names or engage in conversations with fellow residents, diminishing their opportunities to develop meaningful friendships, and contributing to a feeling of disconnection (6, 23). Buckley and McCarthy (24) found similar feelings of disconnection among cognitively intact residents. In their study, residents described a lack of opportunities to engage and build new friendships with fellow residents due to “feeling different” from residents living with dementia. Some even reported difficulties with establishing close relationships with fellow residents due to the lack of common interests and shared hobbies.

Various factors at the organizational level can also contribute to the risk of social isolation, including staff shortage and high turnover (6, 25). Due to constrains in time and resources, healthcare workers often find themselves compelled to prioritize residents’ essential care needs, leaving limited room for addressing their social and emotional needs (6, 11). Moreover, the high staff turnover rate often observed in long-term care facilities can be disruptive to residents’ sense of continuity and familiarity. Having to repeatedly adapt to new caregivers can make it difficult to form lasting connections with the staff, leading residents to rely more heavily on their own families and friends for regular support and care. Similar observations have been made in Norwegian long-term care facilities, where, despite being proficient in delivering essential treatment and care, there is often a shortfall in providing adequate social care (26). For instance, Schönfelder et al. (11) found that care is understood rather instrumentally by most healthcare workers in Norway, usually limited to health-related issues and practical tasks. Social care in form of listening and conversing with the older care recipient is often positioned outside of their professional mandate and treated more like a common courtesy or something that occasionally occurs if there is a surplus of time. Although social care falls within the daily responsibilities of the nursing home staff, the scarcity of time and resources often leads to essential care taking precedence, and social care thus becomes a task for family members and the residents’ closest network.

However, it is not uncommon for residents to experience a sense of disconnection from the broader community as well. Geographical constrains, such as long travel distances to and from the care homes, can impact the frequency of visitations from friends and family. Older residents may also experience difficulties maintaining relationships with their existing network as they might feel that they have little to exchange with friends residing outside the facility (6, 24). Efforts to support the social needs of older residents could be enhanced by focusing on innovative strategies that bridge their connection with the broader community.

### 2.3 Technology use in long-term care

The government in Norway has for a long time recognized the invaluable support families and informal carers provide to both the residents and the healthcare services, and are continuously working toward establishing support measures that can assist informal carers in maintaining the level of social care they provide today (27). One such strategy has been to invest in and promote the use of social welfare technologies to facilitate connection between older residents and their families. Social welfare technologies are technological solutions created to simplify and assist people in connecting with others and is intended to enhance the well-being, quality of life and social inclusion of vulnerable individuals (26). Video call solutions such as Skype, FaceTime and Zoom have frequently been used to help older residents stay connected with friends and family members (28, 29). However, these solutions often require assistance from healthcare staff as most older residents tend to lack the digital competence to operate digital devices on their own (30). Physical and cognitive impairment can further prevent them from using technological solutions, and residents with reduced muscle function may find it particularly difficult to operate handheld devices (3).

Technology’s role in fostering meaningful human connections versus potentially widening the gap between individuals has for long been a highly debated question, especially within the fields of life sciences and social sciences (31). The question is particularly relevant within the context of an aging population and the increasing digitalization of everyday life. While some argue that social technologies foster connections and combat social isolation, especially among the aging population (14, 17, 32–34), critics worry about a digital divide and reduced face-to-face interactions, fearing that digital communication might replace personal visits in long-term care facilities (30, 35). However, research suggests that the main issue is not the technology itself but the lack of solutions tailored to older adults’ needs (17, 36, 37). This study examines the use of Komp – a communication technology specifically tailored to the older demographics needs – and thus adopts an empirical approach to the ongoing debate surrounding technology and social contact. Research on the use of technology among older adults in a nursing home context can yield valuable insights that can shed light on the complex dynamics at play in this debate.

## 3 Materials and methods

### 3.1 Intervention: Komp – a communication technology

**Figure 1:**
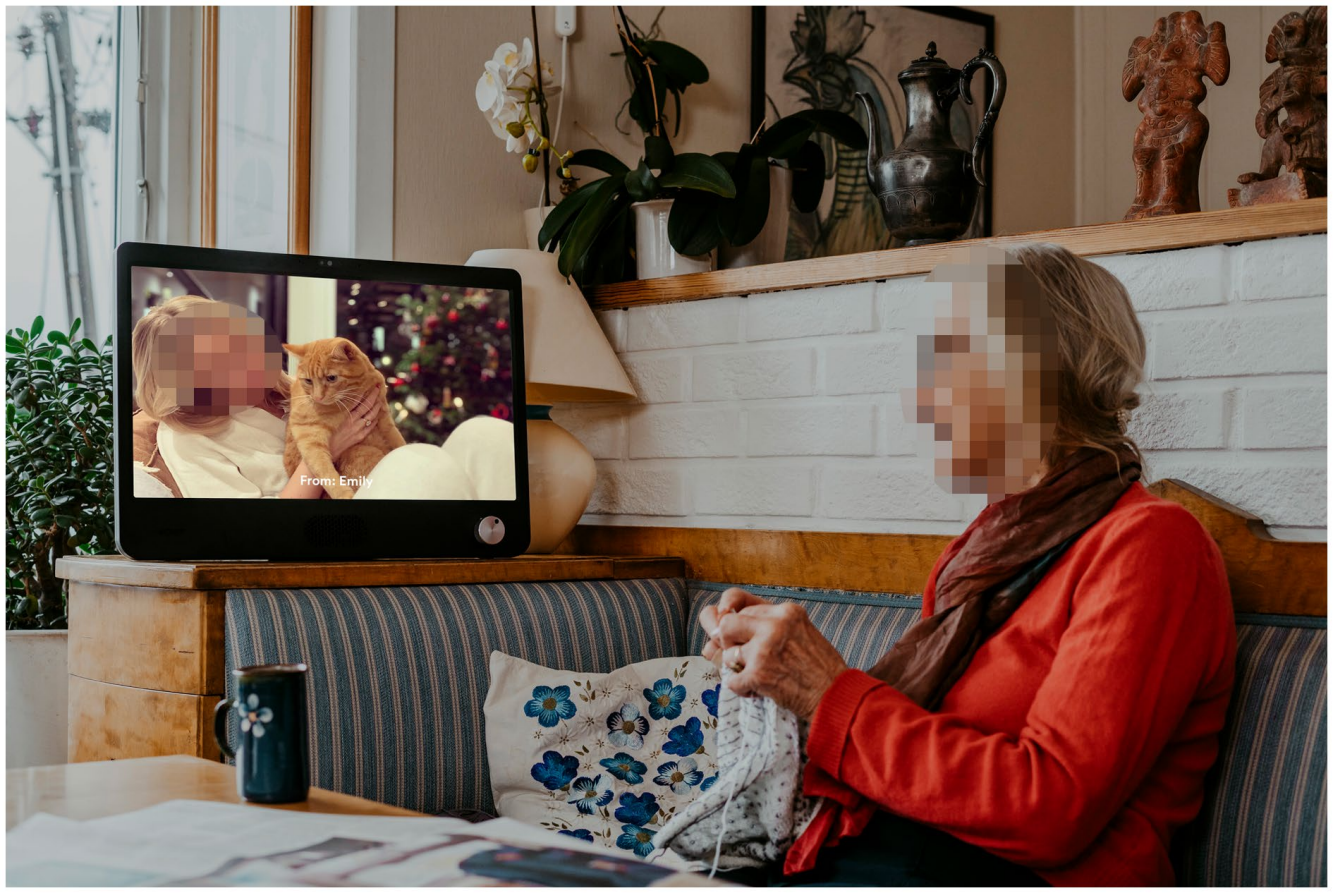
Promotion picture of Komp, used with permission by No Isolation.

This article focuses on the use of a specific communication technology called Komp to facilitate for social contact between nursing home residents and their families. The digital solution consists of two components: A simple screen with one button that turns it on and off and adjusts the volume, and an app that connects to the screen (see Figure 1). Family and friends can use the app on their phones to communicate with the Komp-screen by sending pictures, short messages and make video calls directly to the screen. Komp was created by the Norwegian company No Isolation to combat loneliness and social isolation among older adults by keeping them in touch with their more digitally experienced families. The screen is stripped of all functions assumed to be unnecessary (i.e., touch screens and choices), and enables older users with no digital experience to participate in the everyday lives of their family and friends (38).

Komp aims to facilitate safe and secure communication by only allowing invited users to connect to the screen. Each of the 225 participating nursing home residents received their own Komp-screen which was placed on a flat surface in their own private room. Their relatives were then instructed to connect to the screen through the app on their private phones and they could invite other family members and friends to join the Komp-network as well. Once connected, the residents would be able to receive pictures, short messages and video calls from their families and friends. As long as the screen was turned on, the pictures and short messages would be displayed on the screen, and the video calls would automatically be answered after a countdown of 10 s. If the resident wanted to decline a video call, they would simply have to turn the screen off. Even if the screen was off, the residents would still be notified about receiving new pictures or messages with the help of a small blinking light at the corner of the screen.

Komp has previously been the focus of a few qualitative studies showing mixed results (39–41). While Rasmussen and colleagues (39) found that Komp has the potential to promote digitally mediated family presence for older users that are unfamiliar with modern technology, Badawy and colleagues (40) pointed out that effective use of Komp in care facilities required cooperation between a host of actors, including residents, relatives, and the staff, despite its simple user interface. Another study highlighted Komp’s viability in strengthening established social relationships among the oldest old (41). While the qualitative studies offer valuable insight into Komp’s functioning, this study is one of the few to employ a large-scale quantitative approach to examine the use of a communication technology among older long-term care residents.

### 3.2 Data

This study was part of a larger research and innovation project called “Simple and secure communication between caregivers and recipients” (42), initiated in response to the Covid-19 pandemic. The objective of the current study was to (1) examine whether the communication technology Komp can be used to facilitate social connection and decrease social isolation among nursing home residents, and (2) to examine whether Komp can increase their experience of satisfaction with the social contact. The study uses survey data from a total of 225 nursing home residents from the participating 19 public nursing homes in Oslo, collected over a 14-month period between August 2020 and December 2021. The data collection span, aligning with the pandemic, meant it occurred at a time where alternative solutions to meet the residents’ social needs were highly sought after. The survey data were collected through online surveys at three different waves during this period. The first wave of data, the baseline data (*t_1_*), was collected before Komp was implemented to give an estimate of the frequency and satisfaction with the social contact the residents had with their families before intervention. The second wave of data (*t_2_*) was collected 2 months after Komp was implemented, and the third wave of data (*t_3_*) was collected 6 months after the implementation. The survey data were later combined with a second source of data – a highly detailed weekly datastream collected from each Komp-device – which gives an objective measure of how much Komp was used between the different waves. The datastream gives information about the *frequency* and *type* of content (e.g., the total number of messages, pictures, and video calls) received by the residents on their Komp-screens. To protect the privacy of the users, the datastream does not give insight into the actual messages and images received by each resident on their Komp.

### 3.3 Participants

The participants in this study were the nursing home residents that received a Komp as part of the project. Each nursing home had appointed one main coordinator to oversee the project details and had a small team of staff helping them with the implementation and data collection process. The participants of this study were recruited by the main coordinator from each nursing home who was instructed to recruit individuals who would benefit from using a Komp. Meaning that the premise for being able to participate in the research project and receiving a Komp was that the residents had to have a family member who could connect to the Komp from their own phones or tablets. Furthermore, the participating residents also had to be able to answer the online surveys sent out throughout the project period, either on their own or with the assistance of the nursing staff.

Only residents with a combination of survey answers and datastream information were included in the analysis. This provided us with data from a total of 225 residents. In the overarching project, a total of 338 Komp-devices were allocated to the 19 participating nursing homes, meaning that 163 residents were excluded from this study. Most of residents excluded from this study lacked survey data, usually due to a decline in health and cognition, which made it difficult for them to answer surveys beyond the first wave. As this study looks at the relationship between Komp-use and overall satisfaction with their familial contact, residents who only participated in the first wave before Komp was implemented were naturally not included. In the case of health or cognitive decline, either prior to or after the implementation of Komp, the family members that wanted to continue using Komp were given an exception where they continued answering their own surveys, while the residents were exempted from filling out theirs. This enabled us to still collect important datastream information and survey data from the families, but ultimately reducing the number of participants included in this particular study. Only nine residents out of 163 were excluded for other various reasons. Two residents lacked survey data due to lack of language skills, four residents lacked survey data due to withdrawal of participation, while three passed away soon after the first wave was completed.

### 3.4 Variables

This paper builds on Cornwell and Waites (7) distinction between *social disconnectedness* and *perceived isolation* as two forms of social isolation, and that is why both the quantity and quality of contact is of importance in this study. Social disconnectedness is the lack of social relationships and low levels of participation in social activities and is in this study assessed using questions about the frequency of contact the residents have with their family members. Perceived isolation is a subjective experience of shortfalls in one’s relationships compared to what one would like to have and is measured by how satisfied the residents are with their overall social contact with their family members. The hypothesis of the present study is that increased use of Komp is associated with increased satisfaction with the social contact the residents have with their families.

The dependent variable in this study is the resident’s perceived satisfaction with the social contact they have with their families. This is measured using the question of how satisfied the resident is with the contact they have on a scale from 0 to 10, where 0 means “not satisfied at all” and 10 means “very satisfied.”

The predictor variables measure frequency of contact through Komp, which was assessed in great detail using the datastream collected from each Komp screen. This information is included as two continuous variables. The first variable summarizes the number of messages, pictures and video calls each resident received on their personal Komp in total from the first day of activity (i.e., the day the first picture, message or video call was received on the Komp) until the second wave of the survey was answered (*t_2_*). The second variable summarizes all the messages, pictures and video calls each resident received on their Komp between the time the second survey (*t_2_*) was answered and third survey (*t_3_*) was completed.

A set of important demographic control variables were also included in the analysis. These variables were based on the baseline survey (*t_1_*), which included a set of questions about the residents’ background, including their age, gender, education, number of children, country of birth and which nursing home they belonged to. Education was categorical with the options “No completed education,” “Primary school level,” “Gymnasium/High School,” “Higher education (up to 3 years)” and “Higher education (more than 3 years).” The variable was dichotomized into those who had no education at all (reference) and those who had some level of education. Number of children was also dichotomous with the categories being “no children” (reference) and “one or more children.” The country of birth was dichotomous with the categories being “born in Norway” (reference) and “born in another country.”

Three additional types of communication were included as control variables in one of the models to examine whether the association between social satisfaction and Komp-use is affected by including other forms of communication. The communication types included as control variables were physical contact (e.g., in-person visitations), contact through phone calls, and contact through other digital devices aside from Komp (e.g., computers and tablets). Physical contact and telephone contact was measured using the questions “*How often do you have physical contact with the following people: your children, your grandchildren, your siblings and other family members*” and “*How often do you have telephone contact with the following people: your children, your grandchildren, your siblings and other family members*.” The response options were *1 = Rarely, 2 = A couple times a year, 3 = Once a month, 4 = Several times a month, 5 = Once a week, 6 = Several times a week*, and *7 = Daily*. Both physical contact and telephone contact were turned into two separate index variables: One that measured the total frequency of physical contact with the whole family by combining “children,” “grandchildren” and “siblings and other family members,” and one that measured the total frequency of telephone contact with the whole family by combining “children,” “grandchildren” and “siblings and other family members.” Both measures were included in the analysis as continuous variables. Digital contact was measured using the question “*How often do you have contact with your family using a computer, tablet* etc.*?”* with the response options being *0 = never, 1 = rarely, 2 = occasionally, 3 = often.* This categorical measure is included in the analysis as a dummy variable.

### 3.5 Analysis

Linear multiple regression was carried out to examine the relationship between social satisfaction among the residents and the frequency of contact they had with their families through Komp. The analysis was modeled in three steps: First, the association between social satisfaction and Komp-use was estimated (model 1). In model 2, the demographic control variables – age, gender, education, country of origin, number of children, and which nursing home the participants belong to – was included, since such factors are likely to be related to both the outcome and the main predictor variable. In model 3, in addition to the demographic control variables, other communication types such as physical contact, phone contact and digital contact were also included in the estimates to explore whether these communication types might affect the relationship between social satisfaction and Komp-use.

## 4 Results

Table 1 presents the background characteristics of all the participating nursing home residents in this study. A total of 222 residents responded to the online survey during the first wave/baseline. The three missing respondents were residents who were unable to answer the first survey due to a decline in health but who had recovered in time to continue their participation in the second and/or third wave. The mean age of the total sample at baseline was 86.15, with the youngest participant being 46 years old, and the oldest being 103. Over 70% of all the residents were female, and all but five of the residents reported having some level of education. Over 92% reported being born in Norway, with the remaining 7.21% being born in another country. When asked about number of children that the participants had, over 91% reported having one or more children, while only 8.11% said they had no children.

**Table 1:** Characteristics of the total sample.

	*n*	%
Age		
Mean (SD)	86.15 (10.36)	
Minimum-maximum	46–103	
Gender		
Male	50	22.52
Female	172	77.48
Education		
No completed education	5	2.25
Primary school level	41	18.47
Gymnasium/High school	74	33.33
Higher education (up to 3 years)	50	22.52
Higher education (more than 3 years)	52	23.42
Country of origin		
Born in Norway	206	92.79
Born in another country	16	7.21
Number of children		
None	18	8.11
One or more	204	91.89

Table 2 presents descriptive statistics for the dependent and independent variables, divided by wave. The table shows that most of the residents generally had a high level of satisfaction with their social contact with family but reported having a slightly higher mean score of social satisfaction at the third wave (8.2 on a scale from 0 to 10) than they did at baseline (7.9 on a scale from 0 to 10). According to the information labeled “Communication through Komp,” we can see that the residents on average received 121 pictures, messages and/or video calls from the time they received Komp and up until the second wave was completed. This number had a slight increase 4 months later with a mean score of 146 pictures, messages and video calls received on their Komp-screens. When we look at the frequency of communication through other means, we can see a minor average decrease in physical contact from baseline (3) to the third wave (2.9), meaning that the residents on average reported having physical contact with their families approximately “once a month.” The average communication through the phone and with the help of other digital devices such as a computer or tablets also seem to have decreased slightly between baseline (phone: 3.2, digital devices: 0.7) and the third wave (phone: 2.9, digital devices: 0.6). Table 2 reveals some potentially interesting differences in communication patterns and social satisfaction over time, but only the decrease in telephone communication proved to be statistically significant (*p* = 0.0056). The outcome provides an interesting backdrop for the regression analysis to uncover further insights.

**Table 2:** Descriptive statistics for satisfaction with contact (dependent variable) and frequency of communication (independent variables) for the total sample, by wave.

	Baseline (t_1_)			Second wave (t_2_)			Third wave (t_3_)		
	*n*	Mean (SD)	Range	*n*	Mean (SD)	Range	*n*	Mean (SD)	Range
Social satisfaction	219	7.9 (2.27)	0–10	221	8.1 (1.93)	0–10	180	8.2 (1.97)	0–10
Communication through Komp		–		225	121 (146.1)	0–857	201	146 (278.6)	0–2,576
Physical contact	222	3 (1.00)	1–6	221	2.9 (1.02)	1–6	180	2.9 (0.87)	1–6
Phone calls	222	3.2 (1.39)	1–7	221	3 (1.39)	1–7	180	2.9 (1.26)	1–7
Communication through other digital devices	222	0.7 (1.05)	0–3	221	0.6 (0.99)	0–3	180	0.6 (0.96)	0–3

The dependent variable labeled “social satisfaction” is missing a total of three observations from residents who were unable to, or due to an error, did not answer this specific question during baseline only.

Table 3 presents the regression results for the association between frequency of contact through Komp and the residents’ self-reported level of satisfaction with their overall contact with their families (full table available in the Supplementary Material).

**Table 3:** Social satisfaction and frequency of communication through Komp.

Association between Komp-use and level of social satisfaction reported by residents						
	(1)		(2)		(3)	
	Wave 2	Wave 3	Wave 2	Wave 3	Wave 2	Wave 3
Komp-use (SE)	0.002**	0.001*	0.002*	0.0015**	0.0014	0.001*
	(0.00087)	(0.0005)	(0.00088)	(0.00047)	(0.00086)	(0.00047)
Control variables			Demographic		Demographics and other communication types	
Adjusted R^2^	2.8%	2.5%	15.2%	20%	23.7%	26.4%
Observations	221	180	218	177	218	177

Bivariate regression analysis (1) and multiple regression analysis (2)(3). *p < 0.05; **p < 0.01. Demographic variables included in models 2 and 3: Age, gender, education, country of origin, nursing home and number of children. Other communication types included in model 3: Physical contact, phone contact, other digital devices.

Model 1 shows us the results from a bivariate regression analysis of level of satisfaction and frequency of Komp-use. Model 2 shows us the relationship between level of satisfaction and Komp-use, controlled for age, gender, country of origin, education, nursing home, and whether they have children. Each additional picture, message, or video call received by the residents is associated with being 0.002 scores more satisfied with the overall social contact with their families. This association is adjusted for background variables, with data measured at the second wave.

As we saw in table 2, residents on average received 121 pictures, messages, or video calls from the time they received Komp and up until the second wave was completed. Komp-use is therefore measured in units of 100 going forward to facilitate more accurate interpretation of the coefficient. That means that residents who received 100 pictures, message, and/or video call to their Komp between start-up and the second wave are 0.2 more socially satisfied with the contact on a scale from 0 to 10. When we consider this relationship adjusted for demographic variables after 6 months of use (Wave 3 model 2), the strength is somewhat reduced. Now, an increase in 100 items is associated with being 0.15 points more satisfied with the overall social contact.

Model 3 presents the results from the full model. In this model, other communication types such as physical contact, phone contact and digital contact are controlled for, in addition to the demographic variables from model 2. This model allows for an examination of whether other types of communication influence the relationship between social satisfaction and Komp-use. Model 3 show that the coefficient (b) for Komp-use has a minimal decrease from 0.002 in model 2 to 0.0014 in model 3, 2 months after use. This decrease in the coefficient, although minimal, suggests that other forms of communication might affect the relationship between Komp-use and social satisfaction. When we control for other types of communication in model 3 after 2 months of Komp-use, the associations between Komp-use and social satisfaction are no longer statistically significant. However, a similar decrease can also be seen after 6 months of use in both model 2 and 3, where the association is statistically significant.

## 5 Discussion

This study aimed to assess the potential of the communication technology Komp in fostering social connections and consequently reducing social isolation among nursing home residents. My main hypothesis was that increased use of Komp predicts an increased level of satisfaction. The results from the regression analysis shows a positive and significant relationship between use of Komp and increased social satisfaction, implying that Komp can indeed be a feasible tool for nursing home residents to maintain social connection with their families. Furthermore, the findings showed that the positive impact of Komp on social satisfaction was consistently significant despite the diversity of the sociodemographic aspects, highlighting its applicability and effectiveness among a heterogeneous group.

While this underscores the potential of communication technologies to address social isolation among nursing home residents, it is a particularly encouraging finding given the context of long-term care. Older residents can often face challenges establishing new relationships due to various factors such as declining health, cognition and mobility, limited time to converse with the nursing staff, or simply the lack of common ground and shared interests with fellow residents (6). The literature on the topic thus highlights the importance of assisting residents in maintaining and fostering their existing relationships, which can be challenging without the right tools. There is therefore a need for interventions that can help residents feel connected to the outside world, and the findings of this study suggests that interventions such as Komp have the potential to meet older residents’ social needs by bringing the outside world a little closer. By facilitating regular communication and interaction between residents and their families, Komp can help bridge the physical and emotional distance that often arises when older adults transition to long-term care facilities.

The findings of a positive and significant relationship between use of Komp and increased social satisfaction also aligns with the broader framework of social connectedness, which emphasizes the importance of interactions, relationships, and engagement with others for our overall well-being. Komp enables residents to participate in everyday moments through pictures, text messages and video calls from their families, and provides a sense of presence and belonging from afar. The findings thus indicate that Komp not only has the ability to reduce disconnectedness (lack of contact with others) but also perceived isolation (subjective experience of shortfall in one’s relationships compared to what one would like to have). Moreover, while Komp relies on its simple design and ease of use to appeal to its targeted audience, it also seems to serve a vital perceived usefulness for the older demographic: facilitating contact with their families. Maintaining contact with family members is a significant motivator for older adults to learn new technologies (14). As Komp’s primary objective is to facilitate contact between older relatives and their more digitally experienced family members, the positive findings in this study could partially be attributed to the fact that all participants of this study had a family to connect with.

Neves et al. (17) further highlights the importance for tailored use of communication technologies to fit the older residents’ needs, which is another possible explanation of the positive outcomes observed in this study. However, Badawy et al. (40) found that even tailored and simple technologies like Komp can be difficult to implement in long-term care facilities, as it often creates invisible work for the healthcare workers in an already busy workday. The authors (40) further point out how the implementation of Komp in their study was highly improvised during the Covid-19 pandemic. While Komp was implemented under similar circumstances in the current project, the data was collected in three different waves, meaning that the residents and family members were given more of an adjustment period where they could learn and adjust their use of Komp based on each individual resident’s needs. The adjustment period might be a contributing factor to the positive and significant results in this particular study as it would give families and healthcare workers time to observe what type of pictures or messages the residents preferred and positively responded to.

A noticeable characteristic of the study population that may contribute to Komp’s potential to facilitate social connections is the residents’ age. The average age among the study participants was 86.15 years, with most of them likely having limited prior experience with technology throughout their lives. While one might assume that this lack of familiarity with technology could hinder their ability to adopt new technologies, Komp’s design is specifically intended to be user-friendly for individuals with little to no digital skills. Within this context, the study’s findings align with Komp’s intended design. However, it is important to consider another aspect related to the age diversity within the study population. The youngest of the residents was only 46 years old, suggesting that they likely have had some experience with other technologies such as smartphones and tablets prior to the introduction of Komp. While Komp can be beneficial for many older residents, it may not be the most suitable choice for those who are already skilled at using more advanced digital devices. Similar considerations have been suggested by Rasmussen et al. (39) who found that Komp could potentially be a wrong fit for older adults who are experienced with more advanced technologies such as a smartphone or computer.

The findings of this study can also be an important contribution to the ongoing debate about whether technology can facilitate meaningful human connections or not. The residents’ level of satisfaction in this study implies that they experience the digital contact as satisfying and meaningful, suggesting that the shared digital space created by Komp can foster elements that we typically associate with face-to-face interactions. A possible contributing factor to this experience might be the age-related adjustment to what older residents might expect from their social lives at this point in life. Cornwell and Waite (7) suggest that older people may experience social connectedness and the dimensions of social isolation differently due to having lower expectations for their social lives than other age groups. The residents’ satisfaction with digital contact through Komp may thus be rooted in their unique perspective and adapted expectations regarding social interactions. The potential drawback in this situation however is that the mere presence of Komp in the room can increase the residents’ expectations for social interactions. For some, a black screen or a lack of digital engagement might lead to more disappointment than what they typically would have experiences in the absence of Komp.

### 5.1 Strength, limitations and future research

This is one of the few quantitative studies examining the use of a digital communication technology among older nursing home residents at such a large scale. A total of 225 residents from all public nursing homes in Oslo municipality participated in this study, and the findings of this study thus provides a good basis for generalization. The data for this study was however collected during the Covid-19 pandemic, which means that the social technology was tested out during a time when alternative strategies to facilitate social connection between older residents and their families were highly sought after. While the onset of Covid-19 might have increased the interest in technology-based solutions to social isolation, its usefulness is not limited to such unusual circumstances. The growing social blind spot in eldercare we are experiencing today as a result of high turnover and lack of healthcare workers, as well as the geographical distances between families today show that there will continue to be a need for it beyond the pandemic. Future research may however benefit from looking at the use of communication technologies under more normal circumstances as well. Another strength of this study is that the sample size is diverse in terms of age including both the very young and the very old, capturing some of the variety of a highly heterogenous group. The study however does not include residents without family members or an existing network, potentially excluding those with the highest risk of social isolation. Future research on technology use in long-term care facilities might benefit from including these residents as well.

## 6 Conclusion

This study shed light on the potential of the communication technology Komp to foster social connections and reducing social isolation among nursing home residents. The findings show a positive and significant relationship between the use of Komp and increased social satisfaction across a range of sociodemographic factors, making it a versatile tool in long-term care settings. The study’s findings also contribute to the ongoing debate about the role of technology in fostering human connections. The positive response to Komp among the residents suggests that when designed with the user’s needs in mind, technology can indeed facilitate meaningful social interactions, even for those with limited technological experience. While the diversity in age among participants show variability in Komp’s suitability, such interventions can be crucial in bridging the gap between older residents and the outside world, effectively addressing their unique challenges of social isolation and disconnection from the broader community.

## Data availability statement

The raw data supporting the conclusions of this article will be made available by the authors upon request.

## Ethics statement

The study was approved by Norwegian Centre for Research Data (NSD). The study was conducted in accordance with the local legislation and institutional requirements. Written informed consent for participation in this study was provided by the participants themselves where possible or by the participants’ legal guardians/next of kin.

## Author contributions

SA: Writing – original draft, Writing – review & editing.
