# Supplementary material for: Socially connected while apart: the use of technology to increase social connection between nursing home residents and their relatives
Source: Front Public Health. 2024 Jan 25;12:1296524. doi: 10.3389/fpubh.2024.1296524 (PMC10850371; doi:10.3389/fpubh.2024.1296524)
Supplement: Supplementary file 1 [file Table_1.DOCX]

# **Supplementary material**

**S.1. Full regression results model 2 and model 3**

**Table S.1** Social satisfaction and frequency of communication through Komp. Full multiple regression analysis model 2.

| **Model 2** | | | | |
| --- | --- | --- | --- | --- |
|  | **Wave 2**  **(1-2 months after Komp)** | | **Wave 3**  **(6 months after Komp)** | |
|  | **B** | **p-value** | **B** | **p-value** |
| **Komp-use** | .002 | .022 | .001 | .002 |
| **Gender** (ref=male) | 1.009 | .002 | .20 | .569 |
| **Age** | .001 | .924 | -.01 | .255 |
| **Country of origin** (ref=born in Norway) | -.64 | .193 | .03 | .948 |
| **Education** (ref=no education) | .46 | .604 | -.21 | .848 |
| **Children** (ref=no children) | -.97 | .046 | -.64 | .212 |
| **Long-term care facility** |  |  |  |  |
| 2 | -1.53 | .402 | .76 | .672 |
| 4 | -1.68 | .368 | -.39 | .830 |
| 5 | -1.63 | .386 | - | - |
| 6 | -1.91 | .306 | -1.09 | .568 |
| 7 | -.72 | .695 | 1.81 | .311 |
| 9 | -1.42 | .448 | .66 | .972 |
| 10 | -1.23 | .506 | -.06 | .972 |
| 11 | -2.41 | .194 | -1.83 | .318 |
| 13 | -2.66 | .150 | -1.01 | .574 |
| 14 | -.74 | .689 | .39 | .829 |
| 16 | -2.11 | .259 | 1.51 | .412 |
| 18 | -1.92 | .307 | -.66 | .718 |
| 20 | -1.80 | .358 | .10 | .958 |
| 21 | -.65 | .731 | .84 | .656 |
| 23 | -2.34 | .268 | -2.16 | .279 |
| 24 | -1.66 | .364 | -.07 | .964 |
| 25 | -3.17 | .090 | -1.14 | .535 |
| Constant | 9.26 | .000 | 10.22 | .000 |
| Adjusted R^2^ | 15.2 % | | 20 % | |

**Table S.2** Social satisfaction and frequency of communication through Komp. Full multiple regression analysis model 3.

| **Model 3** | | | | |
| --- | --- | --- | --- | --- |
|  | **Wave 2**  **(1-2 months after Komp)** | | **Wave 3**  **(6 months after Komp)** | |
|  | **B** | **p-value** | **B** | **p-value** |
| **Komp-use** | .001 | .093 | .001 | 0.023 |
| **Physical contact with family** (ref=Rarely) | .18 | .208 | .51 | .011 |
| **Phone contact** **with family** (ref=Rarely) | .39 | .000 | .06 | .647 |
| **Contact through other digital devices** (ref=Never) |  |  |  |  |
| Rarely | -.41 | .260 | .27 | .449 |
| Occasionally | -.32 | .393 | .87 | .060 |
| Often | -.62 | .219 | 1.24 | .018 |
| **Gender** (ref=male) | .88 | .004 | .31 | .372 |
| **Age** | -.001 | .893 | -.01 | .337 |
| **Country of origin** (ref=born in Norway) | -.64 | .187 | -.18 | .716 |
| **Education** (ref=no education) | -.51 | .551 | -.34 | .752 |
| **Children** (ref=no children) | -1.13 | .018 | -1.40 | .010 |
| **Long-term care facility** |  |  |  |  |
| 2 | .02 | .989 | 1.20 | .488 |
| 4 | -.02 | .989 | .30 | .864 |
| 5 | -.17 | .923 | - | - |
| 6 | -.32 | .855 | -.84 | .649 |
| 7 | .69 | .696 | 2.01 | .244 |
| 9 | -.28 | .876 | .04 | .982 |
| 10 | .35 | .844 | -.29 | .868 |
| 11 | -.76 | .667 | -1.17 | .511 |
| 13 | -1.05 | .553 | -.23 | .896 |
| 14 | .80 | .653 | .78 | .658 |
| 16 | -.04 | .980 | 2.10 | .243 |
| 18 | -.47 | .793 | -.04 | .978 |
| 20 | -.10 | .956 | .39 | .833 |
| 21 | .41 | .821 | 1.71 | .350 |
| 23 | -.70 | .732 | -1.85 | .339 |
| 24 | .14 | .934 | .19 | .911 |
| 25 | -1.21 | .506 | -.34 | .850 |
| **Constant** | 6.62 | .005 | 8.37 | .001 |
| **Adjusted R^2^** | 23.7 % | | 26.4% | |
